# Negligible Motion Artifacts in Scalp Electroencephalography (EEG) During Treadmill Walking

**DOI:** 10.3389/fnhum.2015.00708

**Published:** 2016-01-13

**Authors:** Kevin Nathan, Jose L. Contreras-Vidal

**Affiliations:** ^1^Laboratory for Non-invasive Brain-Machine Interface Systems, Department of Electrical and Computer Engineering, University of Houston, HoustonTX, USA; ^2^The Houston Methodist Research Institute, HoustonTX, USA

**Keywords:** electroencephalography, EEG, artifacts, walking

## Abstract

Recent mobile brain/body imaging (MoBI) techniques based on active electrode scalp electroencephalogram (EEG) allow the acquisition and real-time analysis of brain dynamics during active unrestrained motor behavior involving whole body movements such as treadmill walking, over-ground walking and other locomotive and non-locomotive tasks. Unfortunately, MoBI protocols are prone to physiological and non-physiological artifacts, including motion artifacts that may contaminate the EEG recordings. A few attempts have been made to quantify these artifacts during locomotion tasks but with inconclusive results due in part to methodological pitfalls. In this paper, we investigate the potential contributions of motion artifacts in scalp EEG during treadmill walking at three different speeds (1.5, 3.0, and 4.5 km/h) using a wireless 64 channel active EEG system and a wireless inertial sensor attached to the subject’s head. The experimental setup was designed according to good measurement practices using state-of-the-art commercially available instruments, and the measurements were analyzed using Fourier analysis and wavelet coherence approaches. Contrary to prior claims, the subjects’ motion did not significantly affect their EEG during treadmill walking although precaution should be taken when gait speeds approach 4.5 km/h. Overall, these findings suggest how MoBI methods may be safely deployed in neural, cognitive, and rehabilitation engineering applications.

## Introduction

The development of non-invasive mobile brain/body imaging (MoBI) techniques based on active electroencephalography (EEG) synchronized with motion sensing (Makeig et al., 2009; Gramann et al., 2014) and advanced signal processing methods to identify and remove physiological and non-physiological artifacts (Rong and Contreras-Vidal, 2006; Velu and de Sa, 2013; Lau et al., 2014; Urigüen and Garcia-Zapirain, 2015) promise to allow neuroscientists and engineers to investigate the neural dynamics in brain networks during natural (i.e., unconstrained environments) cognition and action.

Recent advances in non-invasive EEG to detect brain activation patterns signaling movement intent during locomotive and non-locomotive tasks (Presacco et al., 2011, 2012; Severens et al., 2012; Bulea et al., 2014; Kline et al., 2014) and during assisted walking using in lower extremity wearable exoskeletons (Wagner et al., 2012; Do et al., 2013; Kilicarslan et al., 2013; He et al., 2014; Seeber et al., 2014) offer the potential to elucidate the cortical contributions to gait and the harnessing of such gait-related neural activity for brain-machine interfaces (BMI) to wearable robots for assistive and therapeutic applications (Venkatakrishnan et al., 2014).

However, little is known about the motor circuits for walking in humans. It is generally agreed that central pattern generators are important in the control of walking; however, supraspinal networks, including the cortex, must be critical as a result of the complexity of locomotive and non-locomotive tasks in activities of daily living. Several studies have shown electrocortical activity coupled to gait cycle phase during treadmill walking (Gwin et al., 2011; Severens et al., 2012), and robotic-assisted treadmill walking (Wagner et al., 2012). That primary motor cortex carries information about bipedal locomotion has been directly proven by the work of Fitzsimmons et al. (2009), who demonstrated that chronic recordings from ensembles of cortical neurons in primary motor (M1) and primary somatosensory (S1) cortices can be used to predict the kinematics of bipedal walking in rhesus macaques.

Neural decoding studies based on scalp EEG have shown that linear and angular kinematics (Presacco et al., 2011, 2012; He et al., 2014) and surface electromyography (EMG) activity can be inferred from scalp EEG during treadmill or robot-assisted walking (He et al., 2014). Moreover, recent studies have deployed neural classifiers based on scalp EEG signals to detect cortical involvement immediately before gait (e.g., gait intention detection; Velu and de Sa, 2013; Jiang et al., 2015) or non-locomotion tasks such as sit-to-stand and stand-to-sit (Bulea et al., 2014). Closed-loop BMI based on EEG have shown the feasibility of using brain waves to control powered exoskeleton for over ground in individuals with spinal cord injury (Kilicarslan et al., 2013) and in robotic-assisted treadmill walking (Do et al., 2013).

Unfortunately, EEG signals are susceptible to physiological and non-physiological artifacts, including motion artifacts, which may compromise the decoding of gait and the interpretation of the neural signals relevant to bipedal locomotion. Methods have been developed to identify and remove such artifacts from the EEG signals (Gwin et al., 2010; Kline et al., 2015; Urigüen and Garcia-Zapirain, 2015), but the efficacy of such methods has been questioned recently (Castermans et al., 2014; Kline et al., 2015). Given that brain activity and the level of artifactual components may vary with walking speed, experimental setup, quality of the instrumentation, tasks, and expertise of the experimenters, published data remains inconclusive. Thus, the purpose of this study was to examine the potential contributions of motion artifacts in scalp EEG during treadmill walking at three different speeds, as in Castermans et al. (2014). The experimental setup was designed according to good measurement practices using state-of-the-art commercial off-the-shelf instruments, and the measurements were analyzed using time-frequency analysis and non-parametric spectral estimation approaches.

## Methods

### Participants

Three able-bodied males and one able-bodied female (ages 26–33) participated in this study. All participants provided voluntary informed consent and performed study procedures that were approved by the Institutional Review Board at the University of Houston.

### Experimental Procedure and Data Acquisition

The experimental protocol followed closely to that reported in Castermans et al. (2014). Participants were instructed to walk on a treadmill at three fixed speeds of 1.5, 3.0, and 4.5 km/h for a minimum of 3-min sessions for each speed. Whole scalp active 64-channel EEG data were collected (battery-operated BrainAmpDC amplifiers with actiCap system, Brain Products GmbH, Munich, Germany) and labeled in accordance with the extended 10–20 international system. EEG data were online referenced to channel FCZ, while electrode impedances were maintained below 10 kΩ. A wireless interface (MOVE system, Brain Products GmbH) transmitted the data to the amplifier, which applied low-pass analog filters set from DC-1000 Hz, and the EEG signals were digitized at 1 kHz using a BrainAmp DC amplifier linked to BrainVision Recorder software version 1.10.

**FIGURE 1:**
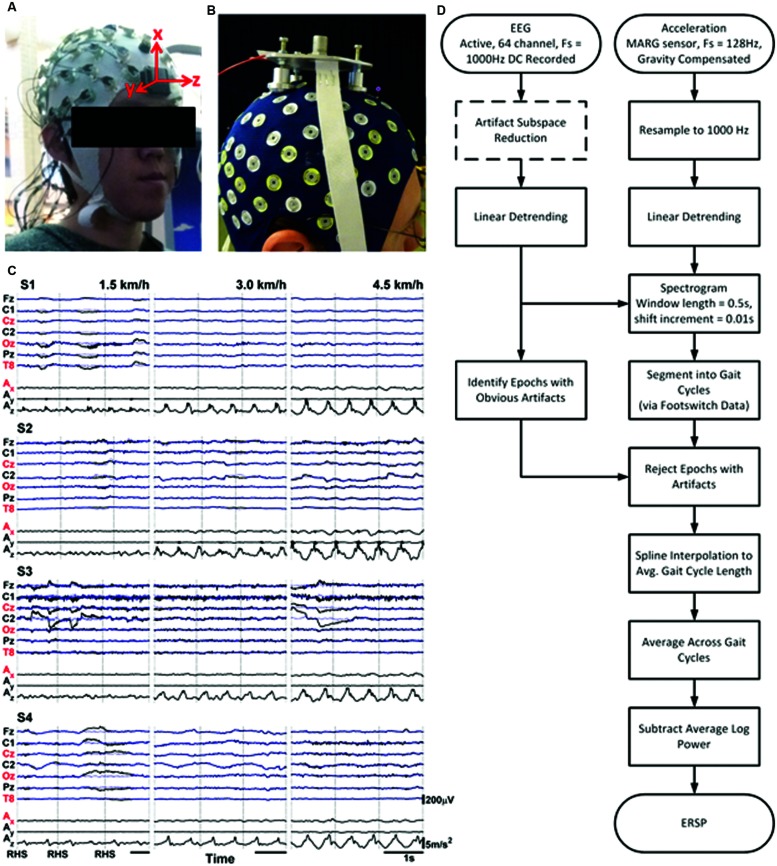
**(A)** Photos of subject and experimental setup showing active EEG cap and triaxial MARG inertial sensor mounted on the forehead (left) and of **(B)** subject and experimental setup from similar protocol in Castermans et al. (2014), reproduced here with permissions from the author and publisher. **(C)** Sample raw EEG and Accelerometer data for three gait cycles for all four subjects and all three speeds. The *x*-, *y*-, and *z*-axes for the accelerometer represent the vertical, mediolateral, and anterior–posterior directions respectively. Traces with red labels indicate channels chosen for further analyses. Blue traces are EEG channels after processing with ASR. Vertical black lines indicate onset of Right Heel Strikes (RHS). **(D)** Flowchart illustrating steps and processes from recording signals to generating ERSPs.

A light-weight wireless Magnetic, Angular Rate, and Gravitational (MARG) sensor (Opal IMU sensors; APDM, Inc.; Portland, OR) was placed on the forehead to record triaxial magnetic, gyroscopic, and acceleration data at a sampling rate of 1280 Hz with an output rate of 128 Hz at 14 bits resolution (bandwidth of 50 Hz) during treadmill walking. The MARG sensor specifications were: weight (<22 g with battery), dimensions (48.4 L × 36.1 W × 13.4 H mm), latency (30 ms), accelerometer range and noise (±6 g; 0.0012 m/s^2^/√Hz), gyroscope range and noise (±2000 deg/s; 0.05 deg/s/√Hz), magnetometer range and noise (±6 Gauss; 0.5 mGauss/√Hz). **Figure 1A** shows a photo of the experimental setup with the subject wearing the EEG cap and Opal sensor. Careful placement of the MARG sensor on the participant’s forehead, as in Kline et al. (2015), ensured no physical interference with the EEG electrodes that could potentially lead to perturbation or distortions of the EEG signals. For comparison, **Figure 1B** depicts the experimental setup used by Castermans et al. (2014) (reproduced here with permissions from the author and original publisher), which may have potentially interfered with the EEG electrode recordings and affected the inertial mass properties of the subject’s head (note that mass of the accelerometer and other technical specifications of this custom setup were not provided in their published manuscript). Flexible contact switches were placed on the heel and toe of both feet to be used as footswitches (FS4 Contact Switch Assembly with DataLOG, Biometrics Ltd., Cwmfelinfach, Gwent, UK), to identify the timing of the heel-strike and toe-off phases of gait at a sampling rate of 1 kHz. EEG, MARG, and footswitch data were time-locked using an external trigger circuit to mark the start and stop of the walking periods. The trigger signal was transmitted wirelessly using the Pololu Wixel RF transmitter/receiver (Pololu Corporation, Las Vegas, NV, USA). **Figure 1C** shows representative raster plots depicting recorded raw EEG (midline channels FZ, CZ, PZ, and OZ as well as channels C1, C2, and T8), and gravity-compensated components of the 3D acceleration (x: gravity; y: toward subject’s left shoulder; z: direction of walking) for the four participants walking in the treadmill at 1.5, 3.0, and 4.5 km/h.

### Signal Processing and Spectrotemporal Analysis

#### Pre-processing

The magnetic, acceleration, and gyroscopic data from the MARG sensor were transformed from the sensor’s reference frame to Earth-fixed frame, and were then used to produce a gravity-compensated acceleration output signal (Noureldin et al., 2013). The corrected acceleration was then resampled to 1 kHz to match the sampling rates of the EEG and footswitch data. Subsequent analysis of EEG signals focused on the CZ, OZ, and T8 channel locations, which cover a broad area of the scalp as in Castermans et al. (2014), and on the vertical (corresponding to our *x*-axis in **Figure 1**) head acceleration, which was shown to have the highest correlation with EEG signals during walking (Kline et al., 2015).

#### Spectral Analysis

The spectra of the three EEG signals and the adjusted (normalized magnitude) acceleration were analyzed by performing a fast Fourier transform (FFT command in Matlab). Default parameters were used (i.e., FFT length was set to the length of the signal rounded up to the next power of 2). The single-sided spectra were analyzed for each channel for each speed and for each subject. These methods were adopted to facilitate comparison with findings from Castermans et al. (2014).

#### Event-Related Spectral Perturbation Analysis

The average deviation from baseline spectral power during the gait cycle, i.e., the ERSP (Makeig, 1993), was computed for each EEG channel and acceleration following the methodology in Gwin et al. (2011). All channels were detrended and a time-frequency analysis was performed on the whole time-series (SPECTROGRAM command in Matlab); a window length of 500 ms was used with an incremental moving window shift of 10ms. The footswitch data was used to segment the full spectrogram into individual gait cycles, marking the times of the right heel strike (RHS), the left toe off (LTO), the left heel strike (LHS), and the right toe off (RTO). The spectrogram for each gait cycle was compared to the time-corresponding raw EEG data; individual epochs that contained large obvious eye or muscle artifacts (based on visual inspection) were rejected (Castermans et al., 2014). The average number of rejected gait cycles for each subject for the 1.5, 3.0, and 4.5 km/h walking speeds were 63, 103, and 88 respectively, out of 145, 184, and 170 total gait cycles; each subject had at least 80 usable gait cycles per walking speed. These remaining gait cycle spectrograms were then interpolated using splines along the time axis to the average gait cycle length for the corresponding subject and speed. With each spectrogram at a common length, the ensemble average time-frequency data were generated by averaging across all gait cycles. The ERSP was computed by subtracting the average log power spectrum for the averaged gait cycle from the log spectrogram at each time point. All steps from pre-processing to ERSP generation are outlined in the flowchart in **Figure 1D**.

#### Wavelet Coherence Analysis

We measured the coherence via a wavelet analysis to analyze frequency correlation between each EEG channel and the acceleration measured by the head-mounted MARG sensor. The raw EEG and acceleration data were similarly detrended, but the time-frequency analysis was performed with the Crosswavelet and Wavelet Coherence package (Grinsted et al., 2004). We used the Morlet wavelet for the mother wavelet and default parameters set to 12 sub-octaves per scale, a minimum scale of twice the sampling interval (2ms) with a maximum scale of one-sixth the signal length times the minimum scale. A Monte Carlo significance test was carried out using 100 iterations.

#### Artifact Subspace Reconstruction

As an ancillary analysis, we repeated the above steps after performing an automated artifact rejection process known as Artifact Subspace Reconstruction (ASR) (Mullen et al., 2013) prior to all other pre-processing steps (see dotted box in **Figure 1D**). ASR is most effective at removing transient, high-amplitude artifacts from eye blinks, muscle bursts, and movement (Bulea et al., 2014); given an input of clean baseline data collected from a minute of standing still, it identifies regions of clean EEG within the data from which it computes an un-mixing matrix based on the geometric median. Principal component analysis is applied to the EEG data in sliding windows, decomposing the data into subspaces, and those subspaces, which deviate from baseline are reconstructed with the un-mixing matrix. ASR can alternatively operate without a separate calibration dataset and automatically search for segments of clean EEG to be used as baseline, but this paper will focus only on the former method. ASR is available as a plug-in for EEGLAB (Delorme and Makeig, 2004), which we used with non-default parameters of a sliding window length of 500 ms, a threshold of three standard deviations for identification of corrupted subspaces (more conservative than the default of five), and without any channel rejection.

## Results

### Frequency Spectra of EEG and Accelerometer

**FIGURE 2:**
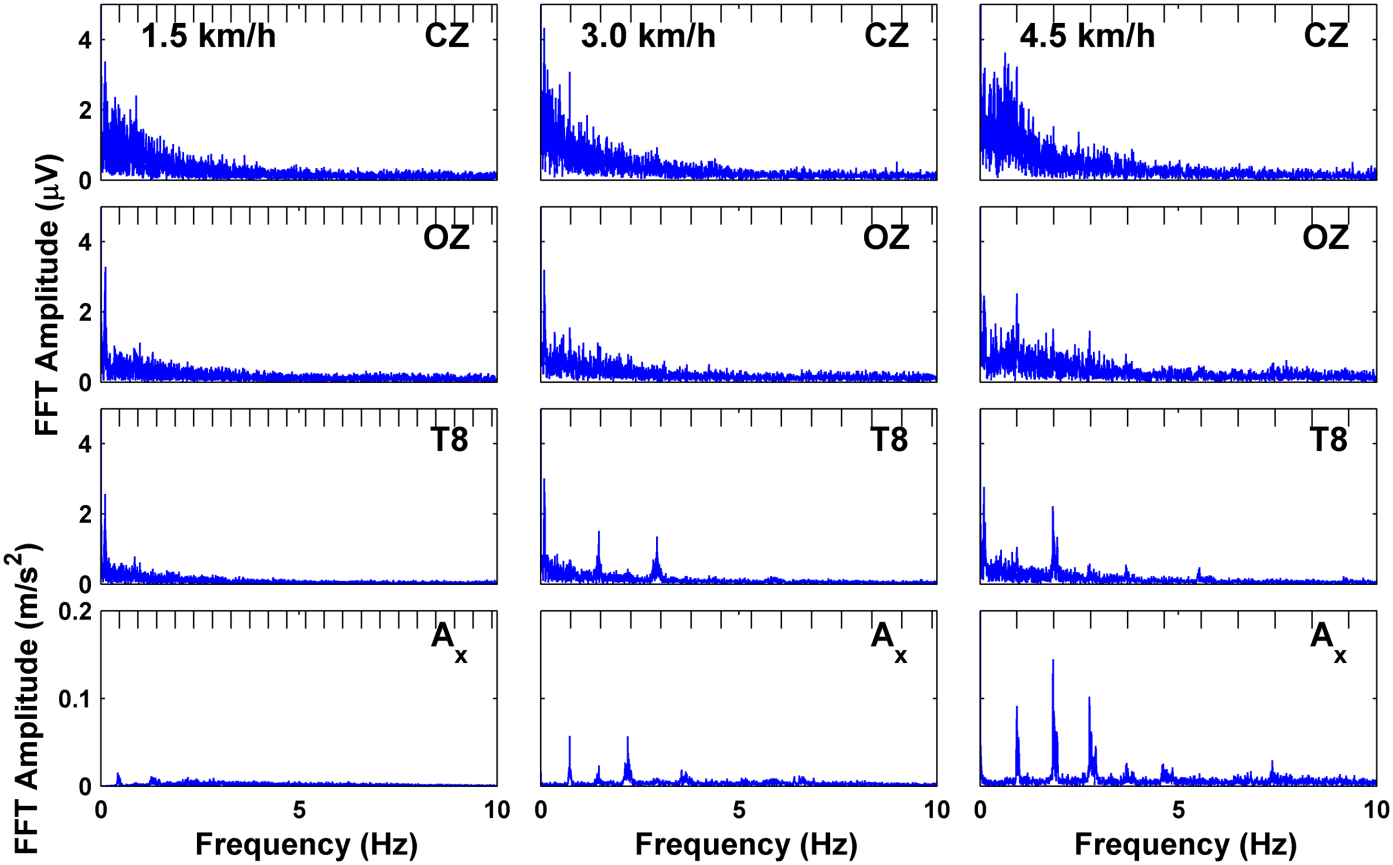
**Fast fourier transform (FFT) of EEG channels and downward acceleration showing frequency spectra for subject S3 at each speed; tick marks on top of plots indicate harmonics of the fundamental stepping frequency**.

Single-sided spectra computed using the fast Fourier transform are shown in **Figure 2** for subject S3 for the three EEG channels (CZ, OZ, and T8), and for the *x*-axis (pointing in the direction of gravity) of vertical head acceleration at three speeds of 1.5, 3.0, and 4.5 km/h. (Sample traces for three full gait cycles for the raw and ASR-processed EEG and acceleration channels are shown in **Figure 1C**). Plots for the other three subjects are included as Supplementary Material as the data is representative across all subjects. The spectra of the EEG channels in our study exhibit commonly found 1/f properties, but absent are large amplitude spikes dominating the spectra which correspond to the stepping frequency of walking (0.47 Hz for 1.5 km/h, 0.76 Hz for 3.0 km/h, 0.93 Hz for 4.5 km/h) or any harmonics thereof, contrary to the findings reported in Figure 1 of Castermans et al. (2014). These spikes are still captured by the accelerometer mounted to the forehead and continue up to 7 Hz, but are only prominent at the two faster speeds. These spikes, albeit greatly reduced in amplitude to the extent that they do not dominate the spectrum, are present in the EEG at the two faster speeds for at least the fundamental harmonic, but are only present at higher harmonics for the fastest speed in OZ and T8, as well as for even harmonics in T8 at the medium speed. The high amplitude spikes do not appear beyond 8 Hz.

### Event-Related Spectral Perturbation Analysis

**FIGURE 3:**
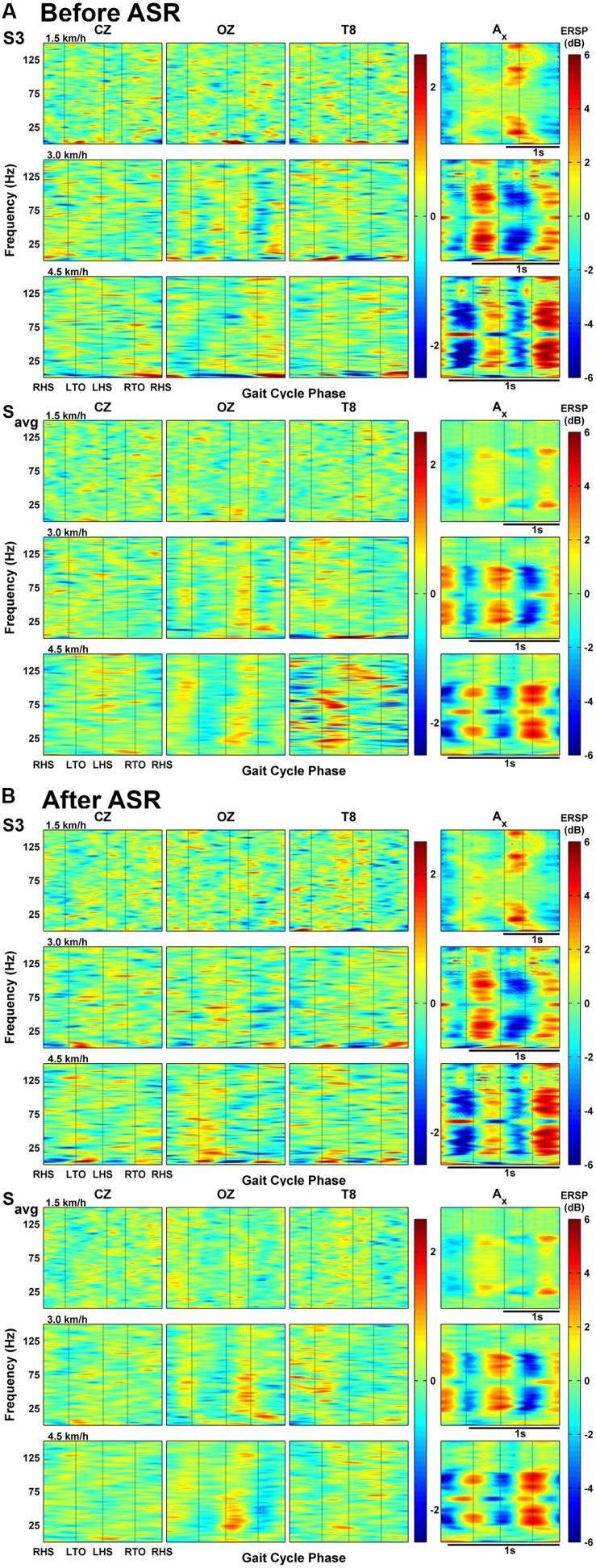
**(A)** Event-related spectral perturbations (ERSPs) of EEG channels and magnitude acceleration averaged across all gait cycles for one subject (S3) and all subjects’ averaged data at each speed. **(B)** ERSP plots of EEG channels and acceleration after processing EEG with Artifact Subspace Reduction.

Average deviations in spectral power throughout the normalized gait cycle are shown in the ERSP plots in **Figure 3** for one individual subject (S3) and for grouped subject data. Individual plots for the other subjects are included as Supplementary Material. The *x*-axis vertical acceleration channel, oriented toward gravity, shows striking intra-stride broadband oscillations, particularly for the two faster speeds (3.0 and 4.5 km/h), spanning continuously (except for an interruption around 60 Hz, likely due to electrical noise) from 20 Hz up to at least 80 Hz. For the individual subject data from S3, we see a decrease in power after the heel strikes followed by a rise during the swing phase (after RTO and LTO). This pattern gets shifted or reversed when averaged across all subjects at 3.0 km/h (and in some cases of other individual subjects at other speeds, see Supplementary Material) with the decrease in power occurring after the swing phases. The oscillations coinciding with gait phase still remain a consistent feature of the acceleration ERSP, but such features are generally absent when comparing with the EEG ERSPs, even when notably reducing the scale of the color bar. Individually, S3 shows some oscillations in power at low frequencies (less than 10 Hz), but these are not seen in the group averaged data.

The above analysis was repeated after applying ASR to the raw EEG data (see **Figure 3B**), with the acceleration ERSPs remaining unchanged. In the group-averaged data, ASR attenuated the changes in spectral power seen in the temporal channels, as alternating regions of dark blue and dark red became negligible. This reduction was not evident in the individual data (S3), and some frequencies showed increased power changes after ASR, particularly in EEG channels at 4.5 km/h.

### Wavelet Coherence Between EEG and Accelerometer Signals

**FIGURE 4:**
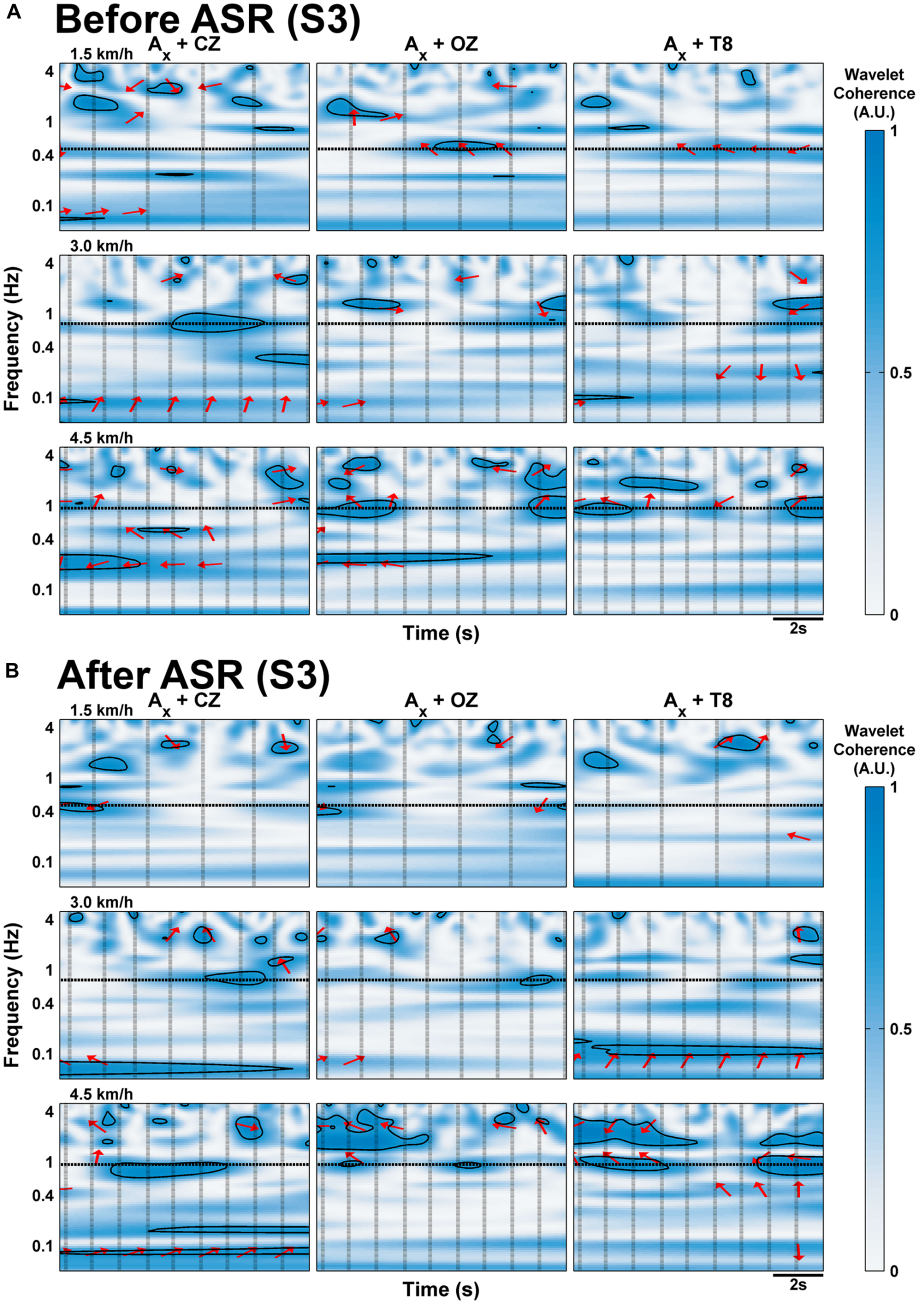
**Wavelet coherence of EEG channels with *x*-axis of acceleration for sample 10 s of walking data for subject S3 at each speed, **(A)** before and **(B)** after processing with Artifact Subspace Reduction.** Frequency is scaled logarithmically on the *y*-axis and is limited to the delta band range of EEG (up to 4 Hz). Vertical black lines indicate onset of RHS; horizontal black lines indicate the frequency of stepping. The arrows indicate the relative phase relationship (in-phase pointing right, anti-phase pointing left, and EEG leading acceleration by 90° pointing straight down), and are only shown for regions with coherence greater than 0.5. Thick black contour lines indicate regions are significant against red noise at the 5% level.

**FIGURE 5:**
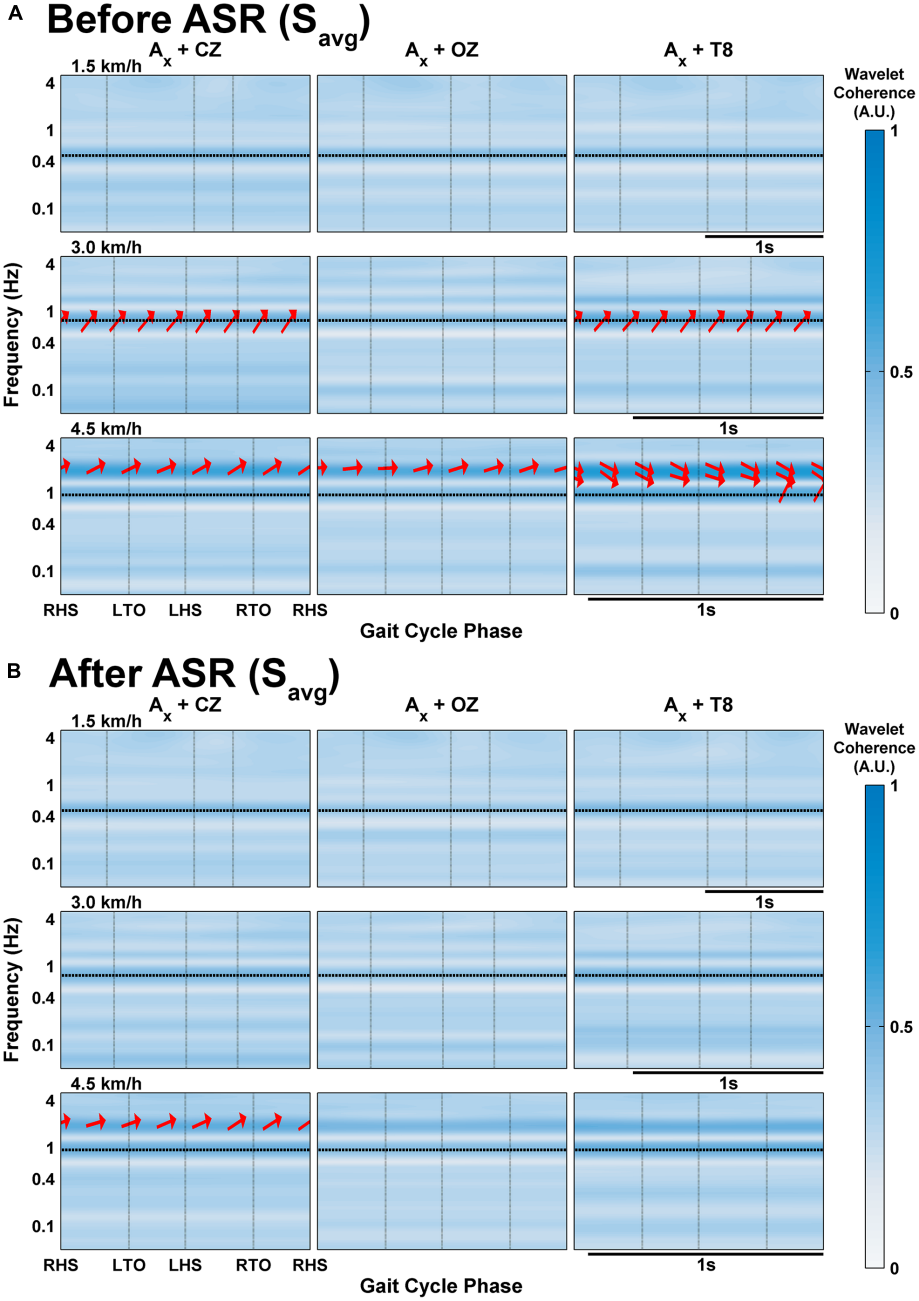
**Wavelet coherence of delta band EEG with *x*-axis of acceleration averaged across all subjects’ gait cycles at each speed **(A)** before and **(B)** after processing with Artifact Subspace Reduction.** Vertical black lines indicate onset of gait cycle phase; horizontal black lines indicate the frequency of stepping. The arrows indicate the relative phase relationship (in-phase pointing right, anti-phase pointing left, and EEG leading acceleration by 90° pointing straight down), and are only shown for regions with coherence greater than 0.5.

We computed the wavelet coherence between the three EEG channels (CZ, OZ, and T8) and the accelerometer, and display continuous time data for one subject (S3, **Figure 4**) and group-averaged data averaged for all gait cycles (**Figure 5**). The Crosswavelet and Wavelet Coherence package (Grinsted et al., 2004) provides not only a time-frequency wavelet analysis, but also gives phase direction and significance levels against Brownian noise (shown as black contour lines). Phase direction is indicated on the figures as red arrows (only shown for coherence values greater than 0.5): arrows pointing right mean completely in-phase relationship between EEG and acceleration with left meaning out-of-phase; upward means a 90° lag of acceleration leading EEG and downward means EEG leading acceleration by 90°. We focus on the slow cortical potentials in the delta band frequencies of EEG (0.1–4 Hz), as these are of primary interest for the decoding of gait kinematics (e.g., Presacco et al., 2011, 2012; Do et al., 2013; Bulea et al., 2014) and display the coherence values for these frequencies on a logarithmic scale.

In the individual subject data (S3 shown in **Figure 4**; additional subjects are included in the Supplementary Materials), regions of significant coherence (at the 5% level determined using Monte Carlo generated noise, marked by thick black boundaries) generally do not span across the gait cycles on a consistent basis, but appear sparsely in small blots. At speeds of 4.5 km/h, long regions of significant coherence span continuously across multiple gait cycles in channels CZ and OZ for S3 with phase angles close to -180° around 0.17–0.27 Hz. Consistent generalizations are hard to discern for all of the subjects, but with the exception of S4 (see Supplementary Materials), significant coherence tends to be seen only at the two faster speeds.

In the group- and gait cycle-averaged data (**Figure 5**), coherence does not seem to vary significantly with time within the averaged gait cycle. The magnitude of coherence in the delta band exceeds 0.5 only at speeds greater than 1.5 km/h. At 3.0 km/h, there is strong coherence (i.e., greater than 0.5) in the CZ and T8 channels between the narrow band of 0.68–0.89 Hz with phase angles varying from 23.9 to 43.4° (mean 33.9°); this band is centered around the stepping frequency for this speed (0.76 Hz), and is likely an artifactual component, especially with the positive phasic relationship. Each channel shows strong coherence at 4.5 km/h between the frequencies of 1.57–2.32 Hz, and also between 0.83 and 1.11 Hz for T8, similar to the coherence at 3.0 km/h; the phase angles were all mostly positive but close to zero for CZ and OZ, calculated to range from -13.6 to 32.7° and -4.3 to 21.9° for the bands in CZ and OZ respectively; for T8, the lower band had positive phase angles between 39.9 and 64.6° and the upper band had negative phase angles toward the end of the gait cycle after the RTO phase from (-28.8)-(-2.02)°.

When ASR was applied, any evidence of delta band coherence disappeared in the averaged group data (**Figure 5B**) except at 4.5 km/h for CZ at the same frequency ranges with the phase angles mostly unaltered. The effects of ASR are quite varied on an individual subject basis: **Figure 4B** shows fewer and smaller regions of significant coherence in S3 after ASR for speeds of 3.0 km/h or slower, except for some low frequency regions below 0.1 Hz, while the opposite effect is seen at the fast walking speed. The rest of the individual subject data and group-averaged data for ASR are included in **Supplementary Figures S3** and **S4**.

## Discussion

### Negligible Effects of Head Motion on EEG Signals During Treadmill Walking

The main finding of this study is that head motion unlikely contaminated the EEG recordings at treadmill gait speeds of 1.5, 3.0, and 4.5 km/hr. This finding is in contrast with a recent study by Castermans et al. (2014), which reported strong harmonics of the frequency of stepping in the Fourier spectra of the EEG (compare **Figure 1** from their paper with **Figure 2** in this study). Some harmonics were present in the spectrum of our acceleration data at walking speeds of 3.0 and 4.5 km/h, but the spectra of the data from active EEG generally do not contain these harmonics to the same extent (with possible exception for channel T8 at 4.5 km/h; T8 and other temporal and peripheral channels are often rejected in EEG decoding studies; Presacco et al., 2011; Bulea et al., 2014; He et al., 2014) while still maintaining the characteristic 1/f power spectrum. The ERSP analyses, which measure the amount of deviation from baseline spectral power during the course of the gait cycle, do not seem to support the claim that motion artifacts are dominant in EEG during walking. If head motion were to affect EEG measurements, one would expect motion-contaminated brain data to show similar ERSP patterns for both the EEG and acceleration data at corresponding walking speeds. Here the *acceleration* ERSP plots show power oscillations of increasing magnitude during treadmill walking that reached the gamma band (30–100 Hz). These oscillations are, however, of smaller magnitude that those reported in Castermans et al. (2014), and more consistent with those reported by Kline et al. (2015) using a similar accelerometer setup. However, in the present study, these oscillations did not appear in the EEG ERSP plots, even with the reduced amplitude scale of -2.5 to 2.5 dB, thus arguing against motion artifacts in the EEG data.

Several discrepancies in the experimental setup and recording techniques may explain these discrepancies. First, it is likely that the experimental setup used in Castermans et al. (2014), reproduced in **Figure 1B** here, distorted the EEG measurements. Specifically, inappropriate setup of the custom accelerometer, may have allowed the accelerometer to transmit shocks to the EEG channels via direct physical contact with the EEG cap and its electrodes. It is widely recognized that most non-physiological artifacts can be reduced by ensuring proper attachment of electrodes or measuring devices (Urigüen and Garcia-Zapirain, 2015). Second, the large inertial mass of the custom accelerometers used in the Castermans study may have contributed to unrealistic and excessive motion (and probably pulling) of the EEG cap and sensors. Moreover, active electrodes used in our study ensure reduced noise from cable movements due to high input and low output impedance, pre-amplification at the scalp level, and cable shielding from electrical noise (Reis et al., 2014). Unfortunately, other technical specifications of the setup, such as mass of the accelerometer and impedances of the EEG electrodes, were not provided in their study, and thus some measurement errors cannot be quantified. Future studies should always include detailed specifications of the sensor and the sensor setup according to good recommended measurement practices in terms of set-up techniques and instrumentation. In particular, care should be taken to minimize interaction between the EEG electrode cap and the connection circuit by isolating the acceleration sensor from the cap. In addition, the weight of the measurement device should be kept as low as is practicable in keeping with current engineering technologies

A recent study by Kline et al. (2015) isolated motion artifact from EEG electrodes during walking by recording the electrical signals picked up after blocking the neural activity with a silicone cap. Their protocol also closely followed that of the present study, including the use of active EEG and an OPAL sensor to monitor head acceleration. The authors were able to characterize pure motion artifact from the electrodes, which they found to have low correlation with the acceleration from the head MARG sensor. Their results lead them to suggest that motion artifact signals are more likely due to electrode cable movement relative to the head as opposed to movement of the head itself. Securing cable motion can be accomplished by using Velcro straps (or similar) to secure cable bundles to the user to minimize cable motion. Cable movements can also be suppressed during MoBI experiments to reduce artifacts by applying a stretchable mesh cap over the electrodes or using a double-layer cap, in which the electrode cables are sandwiched between two layers of fabric (Reis et al., 2014). The Kline study did not, however, include analogous results of recorded EEG data from their setup (except for activity from a mastoid electrode). The present study thus provides valuable information about the potential effects of head motion on EEG recordings.

We extended our analysis to focus on the potential effects of motion artifacts within the delta band (0.1–4 Hz) of EEG, given that this range has been practically used in decoding studies (Presacco et al., 2011, 2012; Do et al., 2013; Bulea et al., 2014; He et al., 2014), based on analysis of wavelet coherence. Coherence has been found between EEG and lower-limb EMG during walking (Petersen et al., 2012) and at delta band frequencies during seated voluntary foot movements (Raethjen et al., 2008). In this situation, high negative-phase coherence (i.e., EEG leading EMG) would support cortical mechanisms for walking; however, high in-phase coherence between the EEG and the accelerometer would suggest mechanical coupling of the electrode leads dominating the EEG signal. However, individual subject data do not show consistent and continuous regions of significant coherence (**Figure 4**, **Supplemental Figure S3**), particularly at lower walking speeds (with the exception of S4). Moreover, because treadmill walking involves continuous and consistent gait motion, we would expect to see the same amount of coherence from one stride to the next if the motion of the EEG setup (e.g., cables going to the amplifiers) affected the EEG signals. The group-averaged data in the current study showed bands of strong coherence (>0.5) for speeds of 3.0 and 4.5 km/h, with phase arrows indicating that the acceleration is leading the EEG signal. At 3.0 km/h, the bands peak at the stepping frequency (∼0.8 Hz) in channels CZ and T8. At 4.5 km/h, there are two bands of strong coherence at 0.97 Hz (average stepping frequency calculated to be 0.93 Hz) and at 1.86 Hz in channels CZ and T8, but only one band for the latter frequency in OZ. Bands of coherence are present at the stepping frequencies at the slowest walking speed but are rather weak in amplitude (∼0.4). This trend is consistent with the individual subject data shown in **Supplement Figure S4**. This could be indicative of artifactual contamination, which would provide some support to previous reports (Gwin et al., 2010; Castermans et al., 2014) of finding motion artifacts at speeds greater than 2.9 km/h. Thus, if the experimenter would like to err on the conservative side, future studies should take special care with cable and electrode motion and electrode impedance to minimize the effects of artifactual components during gait speeds above 3.0 km/h. Prior gait decoding studies have typically measured treadmill gait at rates below 3.0 km/h; the self-selected preferred walking speed of subjects from Presacco et al. (2011) did not exceed 2.4 km/h, and the subjects in this study reported walking at 4.5 km/h to be slightly strenuous. This is in contrast, however, with claims of preferred walking speeds of 1.4 m/s (about 5 km/h) reported in Browning et al. (2006), which was the basis for in-depth artifact analysis of faster walking speeds (1.2 m/s or 4.5 km/h) by Kline et al. (2015). Clinical applications may also be a reason to dictate slower gait speeds for non-able bodied persons. Use of a suitable head accelerometer should likely help to analyze potential motion artifacts in future studies.

### Artifact Subspace Reduction and Other Means of Removing Artifacts

While the main purpose of this study was to understand the extent to which motion artifacts can contaminate raw EEG signals during walking without any processing, we performed our analysis of the EEG data both with and without an automated artifact rejection algorithm known as the ASR method. Mullen et al. (2013) demonstrated that ASR can clean EEG contaminated with eye blink and muscle artifacts without distorting the ERP in an Eriksen Flanker task. Bulea et al. (2014) reported that ASR does not affect decoding from pre-movement epochs (and therefore, free of motion artifacts) of EEG recorded during sit-to-stand transitions, but saw an increase in classification accuracy when decoding from the post-movement epochs when ASR was not used; suggesting that motion artifacts may have been present during these epochs, which contributed to higher decoding accuracies without removing neural signals containing movement-related information. In our analysis, ASR was performed after calibration on motion artifact-free EEG recorded while the subject was standing at rest prior to walking. The algorithm would thereby statistically interpolate any high-variance signal components exceeding a threshold of three standard deviations from the covariance of the calibration data; motion artifacts increasing the variance of the signal would be identified for removal via interpolation (**Figure 1C**). By default, the ASR algorithm uses five standard deviations for the threshold, so our choice of three was a more aggressive criterion. If the algorithm were not given calibration data, it would try to identify clean segments of data within the walking EEG to use for calibration, but this may make it more difficult to identify motion artifacts since their statistics would be too similar to the calibration data.

We found ASR to be most beneficial in attenuating the large amplitude deviations of the ERSP at speeds of 4.5 km/h (**Figure 3**) and in reducing regions of significant coherence (**Figure 5**) at speeds greater than 3.0 km/h; the artifact content at slower walking speeds seems negligible enough to not require additional processing for artifact removal. We note also that sit-to-stand and stand-to-sit actions are likely to be more prone to motion artifacts due to excessive gross movement during these actions. Kline et al. (2015) used artifact removal methods involving a moving average subtraction method and another using Daubechies wavelets; they report similar results in that their methods do not completely clean the signals of artifacts, but do attenuate the peak amplitudes of the ERSP somewhat for the slower walking speeds.

For other MoBI applications that involve excessive movement, including fast treadmill walking or running, there may a stronger tendency for motion artifact to be introduced into the EEG, thereby necessitating optimized artifact removal algorithms to minimize these effects and salvage the EEG signal. Gwin et al. (2010) designed a channel- and component-based artifact removal algorithm that allows for successful performance in a visual oddball discrimination task even while running on a treadmill up to speeds of 6.8 km/h. Their algorithm uses channel-based template regression to remove stride phase-locked artifact followed by an adaptive mixture independent component analysis model (AMICA) to decompose the template-subtracted EEG into spatially static components. Nolan et al. (2010) devised the FASTER algorithm (Fully Automated Statistical Thresholding for EEG artifact Rejection), which detects and removes outliers in various data parameters for EEG time series and independent components. FASTER computes various statistical properties and features for channel data, epoch data, independent components, channels in epoch data, and grand averaged data and removes outliers based on a three *z*-score threshold. FASTER can also detect outlier datasets among aggregated event-related potentials, resulting in a lower baseline variance compared to other supervised methods. Furthermore, O’Regan and Marnane (2013) propose a method to detect artifact contamination using features from both EEG and head-mounted gyroscopes, similar to the setup in this study, which can be used to verify whether or not additional processing is necessary. And while such an algorithm has yet to be tested, Kline et al. (2015) propose a promising method using an EEG cap with certain channels blocked off from electrocortical signals, and using these motion artifact channels to create a template for real-time subtraction. Despite all these concerns over artifacts, Debener et al. (2012) and De Vos et al. (2014) demonstrated that the P300 signal can still be recovered in an auditory oddball task while the subject was walking in a noisy and natural environment, all while using consumer-grade passive EEG equipment. This lends support to the idea that cortical signal is still salvageable even in an ambulatory subject.

This study focused on the possible mechanical artifacts induced in the EEG by the movement of cables due to head motion during treadmill walking. This is not to disregard other potential contributors to artifacts that are not movement-related, but to emphasize that non-physiological artifacts can generally be accounted for and minimized with proper instrumentation and measuring practices (Fatourechi et al., 2007; Urigüen and Garcia-Zapirain, 2015); extraneous sources of noise in non-clinical or laboratory settings can also contribute to artifacts (see review by Sweeney et al., 2012). Physiological sources of artifacts can originate from eye movements and blinks, causing high amplitude deflections (Lins et al., 1993; Croft and Barry, 2000); high frequency electromyographic activity of low and high amplitudes (Willis et al., 1993; Goncharova et al., 2003); electrocardiographic signals producing low amplitude rhythmic spikes (Fisch and Spehlmann, 1999; Sörnmo and Laguna, 2005); and skin perspiration altering electrode impedances causing slow waves in certain channels (Barlow, 1986; Fisch and Spehlmann, 1999). Methods of avoidance, automatic and manual rejection, and removal of these types of artifacts have been extensively reviewed in previous literature (Fatourechi et al., 2007; Urigüen and Garcia-Zapirain, 2015).

## Conclusion

In this study, we analyzed head acceleration data from a forehead-mounted inertial sensor along with active EEG recordings during treadmill walking at three different fixed gait rates. To assess for the potential effects of head motion-related artifacts on the signal quality of the EEG, we analyzed the spectral content of the EEG and acceleration data to inspect for common harmonics in the spectra and computed the ERSPs to measure the deviations from baseline in spectral power across the average gait cycle. We found that the patterns in these measurements are not similar between individual EEG channels and the acceleration, in that strong harmonics in the Fourier spectra and strong spectral gait-synced spectral deviations were found for acceleration but these harmonics were generally absent or negligible in the scalp EEG at gait speeds no faster than 3.0 km/h with minor evidence of motion artifacts in the 4.5 km/h condition in the case of peripheral electrodes. Additional wavelet coherence analysis focusing on the delta band frequency range (0.1–4 Hz) showed a lack of continuous and consistent periods of significant coherence between the head motion and the EEG. Furthermore, comparisons of these findings with results from ASR-cleaned EEG signals suggest that motion artifacts did not likely affect the EEG signals. We primarily found ASR to reduce peak values of spectral deviations in the ERSPs and remove regions of strong coherence for speeds up to 3.0 km/h for the group data. For experiments involving faster walking speeds, ASR may be useful to help mitigate the contamination of motion artifacts. While Kline et al. (2014) demonstrated that moving EEG electrodes certainly create an artifact signal on their own, this study serves to more accurately quantify the extent to which these artifacts may contaminate the EEG signal, with the results showing that they are not as dominant relative to the actual cortical signal recorded by the EEG at slow to moderate walking speeds. Overall, these findings suggest how mobile brain/body neuroimaging techniques methods may be safely deployed, but with caution in regard to appropriate set-ups and common measuring practices, in neural, cognitive, and rehabilitation engineering applications.

## Author Contributions

KN collected the data, performed the analysis, wrote the manuscript, and created the figures for the paper. JV supervised the experiment, provided suggestions for the analysis, and reviewed the manuscript.

## Conflict of Interest Statement

The authors declare that the research was conducted in the absence of any commercial or financial relationships that could be construed as a potential conflict of interest.

## Supplementary Material

**FIGURE S1 | Fast fourier transform (FFT) of EEG channels and downward acceleration showing frequency spectra for each subject at each speed.**

**FIGURE S2 | (A)** Event-related spectral perturbations (ERSPs) of EEG channels and magnitude acceleration averaged across all gait cycles for each subject at each speed. **(B)** ERSP plots of EEG channels and acceleration after processing EEG with Artifact Subspace Reduction.

**FIGURE S3 | Wavelet coherence of EEG channels with x-axis of acceleration for sample 10 s of walking data for each subject at each speed, **(A)** before and **(B)** after processing with Artifact Subspace Reduction.** Frequency is scaled logarithmically on the *y*-axis and is limited to the delta band range of EEG (up to 4 Hz). Vertical black lines indicate onset of Right Heel Strikes (RHS); horizontal black lines indicate the frequency of stepping. The arrows indicate the relative phase relationship (in-phase pointing right, anti-phase pointing left, and EEG leading acceleration by 90° pointing straight down), and are only shown for regions with coherence greater than 0.5. Thick black contour lines indicate regions are significant against Brownian noise at the 5% level.

**FIGURE S4 | Wavelet coherence of delta band EEG with *x*-axis of acceleration averaged across all gait cycles for each subject at each speed **(A)** before and **(B)** after processing with Artifact Subspace Reduction.** Vertical black lines indicate gait cycle phase; horizontal black lines indicate the frequency of stepping. The arrows indicate the relative phase relationship (in-phase pointing right, anti-phase pointing left, and EEG leading acceleration by 90° pointing straight down), and are only shown for regions with coherence greater than 0.5.
