# Supplementary figures and images for: Negligible Motion Artifacts in Scalp Electroencephalography (EEG) During Treadmill Walking
Source: Front Hum Neurosci. 2016 Jan 13;9:708. doi: 10.3389/fnhum.2015.00708 (PMC4710850; doi:10.3389/fnhum.2015.00708)

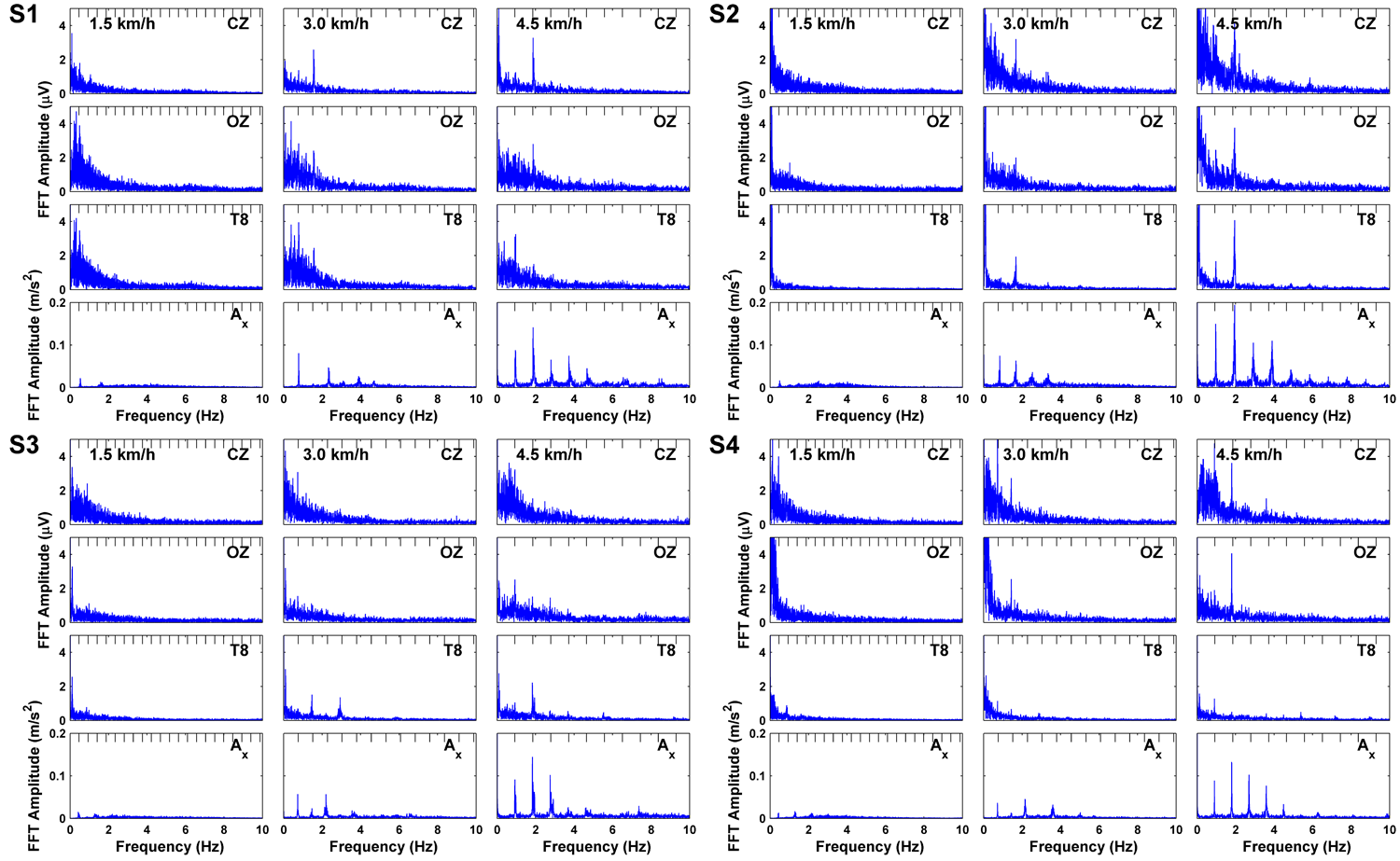

Supplement: Supplementary file 1 [file Image_1.TIF]

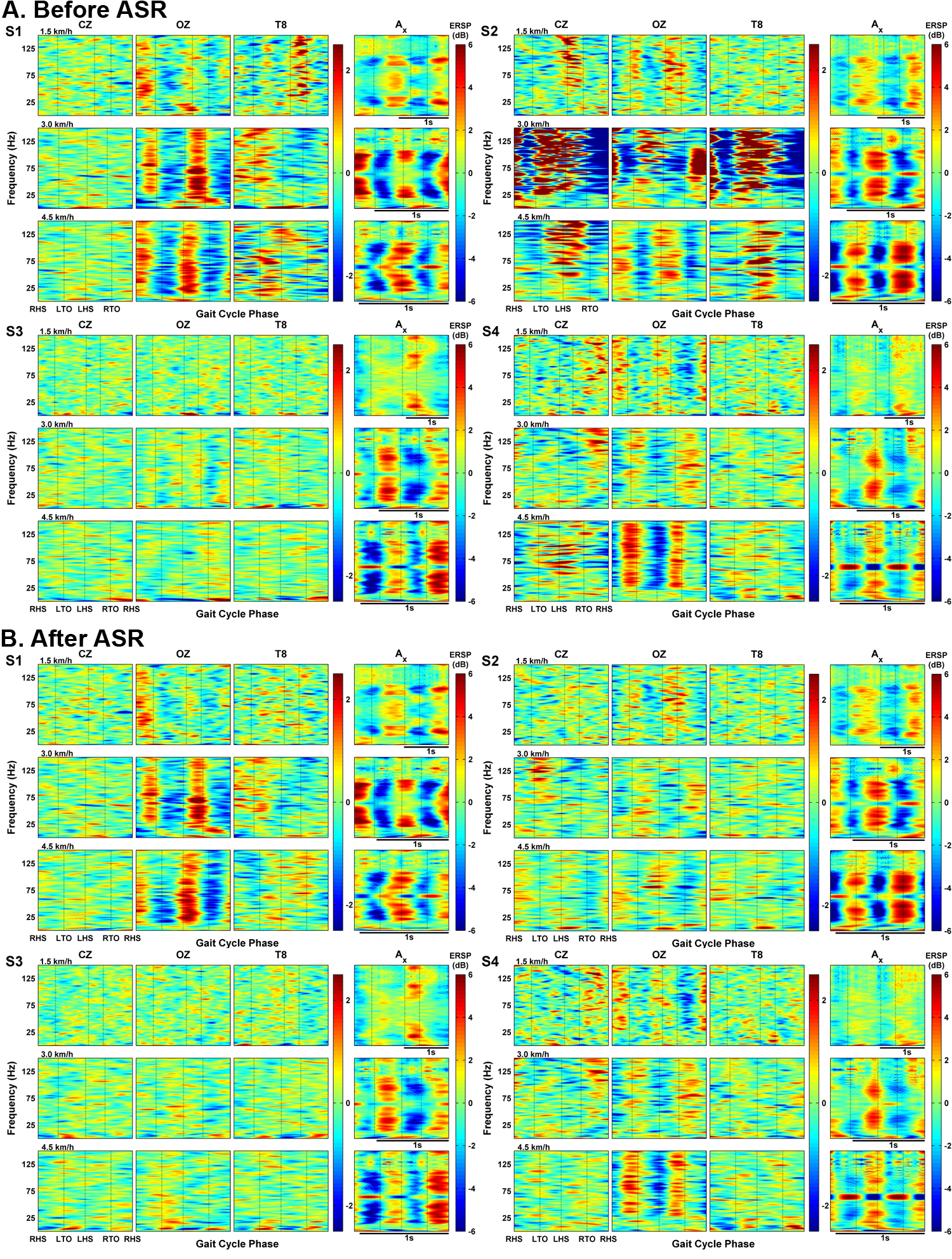

Supplement: Supplementary file 2 [file Image_2.TIF]

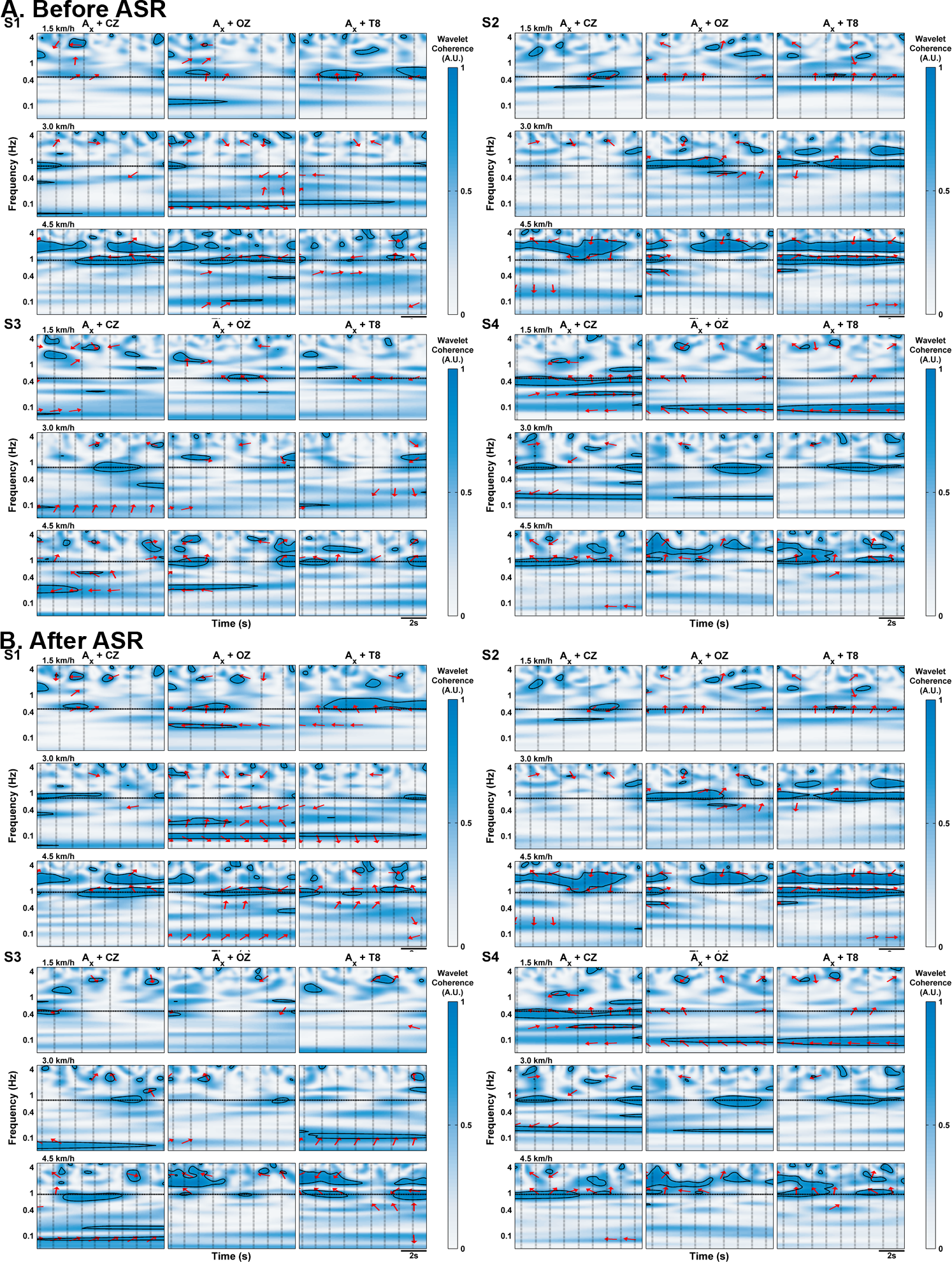

Supplement: Supplementary file 3 [file Image_3.TIF]

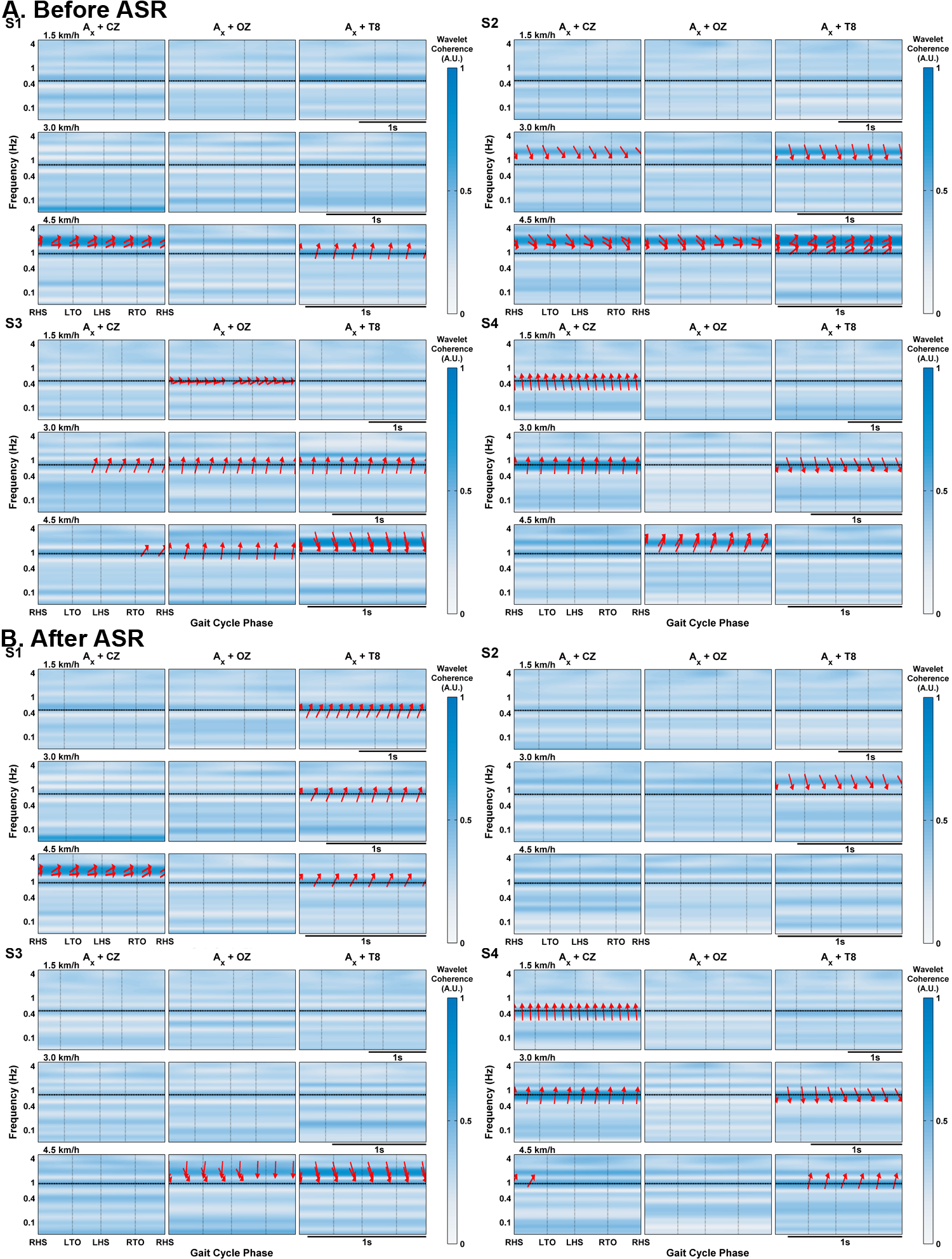

Supplement: Supplementary file 4 [file Image_4.TIF]
